# Prognostic Value of Biomarkers in Cancer Patients Treated With Immune Checkpoint Inhibitor Therapy

**DOI:** 10.1016/j.jacadv.2025.102022

**Published:** 2025-07-31

**Authors:** Christopher Mann, Ulrike Pailer, Andreas Spannbauer, Fardin Hamidi, Aliza Veronika Braser, Martin Hülsmann, Christian Gerges, Michael Gottsauner-Wolf, Mariann Gyöngyösi, Markus Raderer, Martin Riesenhuber, Christian Hengstenberg, Jutta Bergler-Klein, Thomas A. Zelniker

**Affiliations:** a Division of Cardiology, Medical University of Vienna, Vienna, Austria; b Vienna Healthcare Group, Vienna, Austria; c Division of Clinical Oncology, Medical University of Vienna, Vienna, Austria; d Comprehensive Center of Cardiovascular Medicine, Medical University of Vienna, Vienna, Austria

**Keywords:** biomarkers, cardiotoxicity, cardiovascular hospitalization, immune checkpoint inhibitors, myocarditis, risk stratification

## Abstract

**Background:**

Immune checkpoint inhibitors (ICIs) have improved outcomes for several malignancies, but cancer patients face an increased risk of cardiovascular disease due to shared risk factors, similar biological mechanisms, and cardiotoxic side effects of therapy. Effective risk stratification strategies are urgently needed.

**Objectives:**

This study explored the association between the biomarkers with the risks of acute cardiovascular hospitalizations and death in cancer patients receiving ICI therapy.

**Methods:**

We used electronic health records of patients treated with ICI who had available baseline N-terminal pro-B-type natriuretic peptide (NT-proBNP) levels at Vienna General Hospital between January 2017 and July 2022. The primary outcome of interest was the composite of acute cardiovascular hospitalization heart failure or death. Cox regression models were adjusted for age, sex, estimated glomerular filtration rate, diabetes, coronary artery disease, heart failure, hypertension, atrial fibrillation, C-reactive protein, and low-density lipoprotein cholesterol.

**Results:**

Among 550 patients (35% female, age 65 years), median NT-proBNP levels were 272 pg/mL (Q1-Q3: 102-742), with 388 patients (71%) having levels ≥125 pg/mL. Over a median follow-up of 67 weeks, 190 patients (35%) died, and 103 cardiovascular hospitalizations (most commonly due to stroke, coronary artery disease, and heart failure) occurred in 76 patients (14%), resulting in a primary composite endpoint rate of 46.7 events per 100 patient-years. After multivariable adjustment, NT-proBNP remained independently associated with an increased risk of cardiovascular hospitalization and death (adjusted HR for 1-SD increase in log-transformed biomarker: 1.29; 95% CI: 1.06-1.33).

**Conclusions:**

These data highlight NT-proBNP levels as a valuable marker for identifying cancer patients at increased risk for cardiovascular events during ICI therapy.

The advent of immune checkpoint inhibitors (ICIs) has dramatically transformed cancer therapy, providing new hope for patients with various malignancies.^1^ ICIs have been shown to improve survival rates across several cancer types by activating the body's immune system to recognize and destroy cancer cells.^2^ However, this promising innovation is not without challenges, as ICIs are also associated with a wide range of immune-related adverse events that can affect multiple organ systems, including the cardiovascular system.^3^ The cardiovascular toxicities linked to ICIs are complex and potentially severe, with documented cases of myocarditis, pericarditis, arrhythmias, and vasculitis in ICI-treated patients.^4^, ^5^, ^6^, ^7^, ^8^ Emerging evidence suggests that ICIs may also accelerate atherosclerosis, aggravating the cardiovascular risk profile further.^9^ This concern is particularly critical due to the high prevalence of cardiovascular disease in cancer patients, who often share common cardiovascular risk factors, including age, smoking, and other lifestyle-related variables.^10^, ^11^, ^12^, ^13^ The growing population of patients receiving ICIs and the expanding indications for their use further amplify this increased cardiovascular risk. To mitigate these risks, it is essential to ensure regular cardiovascular assessments and identify strategies for risk stratification and early intervention protocols.

In diverse oncological populations, cardiac biomarkers—particularly N-terminal pro-B-type natriuretic peptide (NT-proBNP) and high-sensitivity troponin T (hsTnT)—have shown promise as indicators of cardiovascular events and mortality risk.^14^, ^15^, ^16^, ^17^, ^18^ In alignment with this, the Council on Cardio-Oncology of the European Society of Cardiology (ESC), the 2022 ESC Cardio-Oncology Guidelines, and the clinical practice guidelines of the European Society for Medical Oncology have highlighted the importance of cardiac biomarkers as risk stratification tools prior to the start of ICI treatment.^19^, ^20^, ^21^ However, despite these recommendations, the evidence supporting the use of cardiac biomarkers for risk stratification in ICI-treated patients remains limited and inconclusive.^16^^,^^22^^,^^23^

In the present study, we aimed to evaluate the associations of cardiac biomarkers with the risk of acute cardiovascular hospitalizations and death in ICI-treated patients.

## Methods

### Study population

In this study, all cancer patients who were treated with ICI and had available NT-proBNP concentrations at Vienna General Hospital and Medical University of Vienna, a tertiary academic center, between January 2017 and July 2022 were included. Patients were identified through a comprehensive search of electronic health records, and patients’ demographics, medical history, and laboratory measurements were collected. The presence of cardiovascular disease was ascertained using the International Classification of Diseases codes, as documented in the patients’ discharge summaries (Supplemental Methods).

This study adhered to the principles of the Declaration of Helsinki and received approval from the local ethics committee of the Medical University of Vienna (EK 2249/2018).

### Laboratory measurements

All laboratory measurements were performed at the central clinical laboratory of Vienna General Hospital (Medical University of Vienna). NT-proBNP concentrations used in this study were measured within 3 months prior to initiating ICI therapy using the Roche Diagnostics Cobas immunoassay. As previously reported,^24^ the assay's detection limit was 3 pg/mL, and the analytical range was between 5 and 35,000 pg/mL. The intra-assay imprecision profile was 17.2 pg/mL for a mean value of 1,014 pg/mL with a coefficient of variation of 1.7%. HsTnT concentrations were measured with immunoassays on the cobas e 601 (Roche Diagnostics). The limit of quantitation of the hsTnT assay is 6 ng/L, and the 99th percentile upper reference limit is 14 ng/L. If multiple measurements were available, the value closest to the first ICI administration was selected. We excluded patients with documented acute cardiovascular events at the time of biomarker measurement to mitigate the potential influence of acute conditions on biomarker levels.

### Outcomes of interest

The primary outcome of this study was a composite endpoint of death or acute cardiovascular hospitalization. Secondary outcomes included the individual components of the primary composite endpoint. The cause and date of death were obtained from the national death registry, and instances of cardiovascular hospitalizations were identified using the Vienna Healthcare Group hospitalizations database (“Wiener Gesundheitsverbund”).

### Statistical analysis

Continuous data are shown as median (IQR), and categorical variables as counts and proportions. Kaplan-Meier (KM) event rates at 12 and 24 months were compared using the log-rank test. NT-proBNP and hsTnT were modeled as a continuous standardized log-transformed variable, as well as using quartiles and an a priori threshold of ≥125 pg/mL (as suggested by Heart Failure Association and the International Cardio-Oncology Society) for NT-proBNP and ≥14 ng/L for hsTnT (reflecting the 99th percentile upper reference limit for the hsTnT assay).^20^^,^^25^^,^^26^ Cox regression models adjusted for age, sex, estimated glomerular filtration rate, diabetes, coronary artery disease, heart failure, hypertension, atrial fibrillation, C-reactive protein, and low-density lipoprotein cholesterol were used to assess the relationship between NT-proBNP, hsTnT, and the outcomes of interest. The results are presented as HR and the respective 95% CI. Schoenfeld residuals confirmed that the proportional hazards assumptions were not violated when using Cox modeling. All *P* values were based on 2-sided tests and were considered statistically significant at *P* < 0.05. As this was an exploratory analysis, no adjustment for multiple testing was performed. All statistical analyses were done using R (version 4.2.2, R Foundation for Statistical Computing).

## Results

### Study population

In total, 1,142 patients with cancer who received ICI between January 2017 and July 2022 were identified. Five hundred fifty (48%) patients had available NT-proBNP levels and were included in the present analysis. Patients with available NT-proBNP concentrations vs those without tended to be older (65 vs 63 years, *P* = 0.085), were more likely to be male (65 vs 57%, *P* = 0.005), have atrial fibrillation (4.4% vs 0.7%, *P* < 0.001), heart failure (1.5% vs 0.3%, *P* = 0.056), and coronary artery disease (5.6% vs 2.2%, *P* = 0.003) (Supplemental Table 1), but had similar event rates of acute cardiovascular hospitalizations and death (KM event rates at 12 months: 29% vs 32%, *P* = 0.52).

Among the 550 included patients, the median follow-up was 66.9 weeks (Q1-Q3: 32.5-116.8 weeks). The median estimated glomerular filtration rate was 81 mL/min/1.73 m^2^ (Q1-Q3: 56-112 mL/min/1.73 m^2^). Thirty-one (5.6%) patients had coronary artery disease, 117 (21%) had hypertension, and 71 (13%) had diabetes (Table 1). Malignant neoplasms of the respiratory and intrathoracic organs (n = 130, 24%) were the most prevalent type of cancer, followed by malignant neoplasms of the urinary tract (n = 79, 14%), melanoma (n = 74, 13%), and digestive organs (n = 57, 10%). The majority of patients were treated with pembrolizumab (n = 303, 55%), followed by nivolumab (n = 184, 33%), atezolizumab (n = 79, 14%), and ipilumab (n = 71, 13%). Twenty-one (3.8%) patients had more than 1 cancer diagnosis, and 63 (11.4%) received treatment with more than 1 ICI. Detailed information regarding cancer diagnosis and ICI treatment can be found in Supplemental Tables 2 and 3, respectively.

**Table 1:** Baseline Characteristics

	Overall (N = 550)	NT-proBNP		
		≥125 pg/mL (n = 388)	<125 pg/mL (n = 162)	*P* Value
Age, y	65 (56, 73)	68 (59, 75)	58 (52, 65)	<0.001
Female, n (%)	192 (35%)	137 (35%)	55 (34%)	0.82
Diabetes mellitus, n (%)	71 (13%)	52 (13%)	19 (12%)	0.60
Hypertension, n (%)	117 (21%)	89 (23%)	28 (18%)	0.24
Atrial fibrillation, n (%)	24 (4.4%)	24 (6.2%)	0 (0%)	0.001
Heart failure, n (%)	8 (1.5%)	8 (2.1%)	0 (0%)	0.11
Coronary artery disease, n (%)	31 (5.7%)	29 (7.5%)	2 (1.3%)	0.004
NT-proBNP, pg/mL	272 (102, 745)	480 (253, 1,101)	58 (36, 81)	<0.001
hsTnT available, n (%)	305 (56%)	233 (60%)	72 (45%)	0.001
hsTnT, ng/L	16 (10, 31)	20 (12, 33)	10 (7, 15)	<0.001
hsTnT ≥14 ng/L, n (%)	185 (61%)	162 (70%)	23 (32%)	<0.001
eGFR, mL/min/1.73 m^2^	81 (56, 112)	81 (55, 119)	81 (60, 102)	0.58
CRP, mg/dL	1.1 (0.3, 3.3)	1.3 (0.4, 3.9)	0.6 (0.2, 1.9)	<0.001
HbA1c, %	5.70 (5.30, 6.10)	5.60 (5.35, 6.10)	5.70 (5.30, 6.00)	0.87
LDL-C, mg/dL	95 (66, 122)	88 (61, 110)	113 (88, 137)	<0.001

Continuous data are reported as median (IQR).

CRP = C-reactive protein; eGFR = estimated glomerular filtration rate; HbA1c = Hemoglobin A1c; hsTnT = high-sensitivity troponin T; LDL-C = low-density lipoprotein cholesterol; NT-proBNP = N-terminal pro-B-type natriuretic peptide.

### Biomarker concentrations

The median NT-proBNP levels were 272 pg/mL (102-745 pg/mL), and 388 (70.5%) patients had NT-proBNP levels ≥125 pg/mL. Patients with NT-proBNP levels ≥125 pg/mL tended to be older (68 vs 58 years, *P* > 0.001) and were more likely to have atrial fibrillation (6.2% vs 0%, *P* = 0.001), heart failure (2.1% vs 0%, *P* = 0.11), and coronary artery disease (7.5% vs 1.3%, *P* = 0.004). Moreover, hsTnT concentrations were available for 305 (56%) patients. The median hsTnT levels were 16 ng/L (10-31 ng/L), and 185 (61%) had hsTnT levels >14 ng/L (Table 2).

**Table 2:** Baseline Characteristics Stratified by an hsTnT Cutoff of 14 ng/L

	Overall (N = 305)	hsTnT	
		>14 ng/L (n = 185)	<125 pg/mL (n = 162)
hsTnT, ng/L	17 (10, 31)	27 (18, 39)	8 (7, 10)
Age, y	67 (58, 74)	70 (63, 76)	61 (53, 69)
Female, n (%)	98 (32%)	51 (28%)	47 (39%)
Diabetes mellitus, n (%)	47 (15%)	34 (18%)	13 (11%)
Hypertension, n (%)	81 (27%)	57 (31%)	24 (20%)
Atrial fibrillation, n (%)	22 (7.2%)	19 (10%)	3 (2.5%)
Heart failure, n (%)	6 (2.0%)	5 (2.7%)	1 (0.8%)
Coronary artery disease, n (%)	27 (8.9%)	22 (12%)	5 (4.2%)
NT-proBNP, pg/mL	393 (136, 1,071)	660 (233, 1,573)	177 (62, 481)
NT-proBNP ≥125 pg/mL, n (%)	233 (76%)	162 (88%)	71 (59%)
eGFR, mL/min/1.73 m^2^	85 (62, 123)	95 (71, 152)	76 (53, 94)
CRP, mg/dL	1.1 (0.3, 3.5)	1.4 (0.4, 3.7)	0.7 (0.2, 2.6)
HbA1c, %	5.60 (5.30, 6.00)	5.60 (5.30, 6.10)	5.70 (5.30, 5.88)
LDL-C, mg/dL	96 (69, 121)	90 (66, 114)	101 (77, 125)

Continuous data are reported as median (IQR).

Abbreviations as in Table 1.

### Incidence and causes of acute cardiovascular hospitalizations and death

During the follow-up period, there were 103 cardiovascular hospitalizations in 76 (13.8%) patients (Supplemental Figure 1), and 190 (34.5%) patients died. Overall, there were 46.7 events per 100 patient-years of the primary composite endpoint, with a corresponding 12-month KM event rate of 29%.

**Figure 1:**
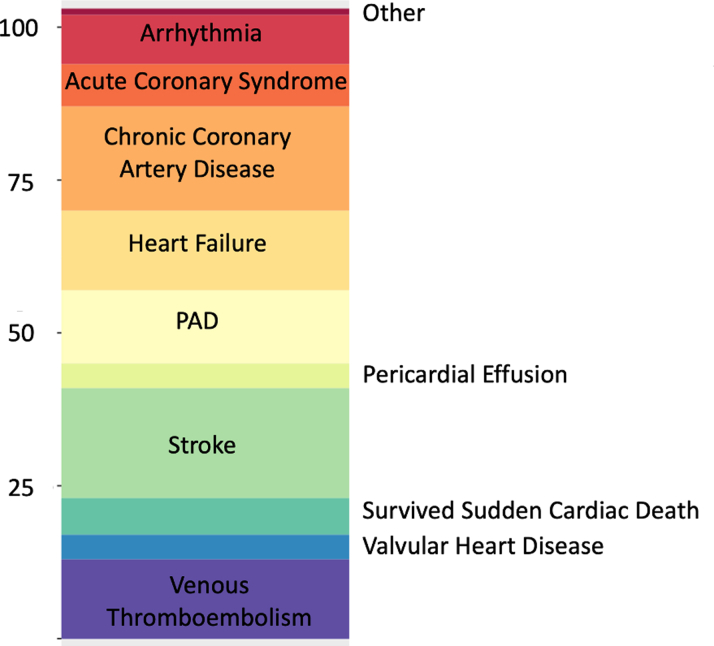
Causes of Cardiovascular Hospitalizations PAD = peripheral artery disease.

Stroke was the most frequent cause for acute cardiovascular hospitalization (n = 18, 18.2%), followed by chronic coronary artery disease (n = 17, 17.2%), heart failure (n = 13, 13.1%), venous thromboembolism (n = 13, 13.1%), peripheral artery disease (n = 12, 12.1%), and acute coronary syndrome (n = 7, 7.1%) (Figure 1). The 13 heart failure hospitalizations occurred in 7 unique patients, none of whom had a documented history of heart failure at baseline. Death certificates predominantly cited cancer as the cause of death, accounting for 97.8% of cases.

### Relationship between cardiac biomarkers and cardiovascular events in ICI-treated patients

**Figure 2:**
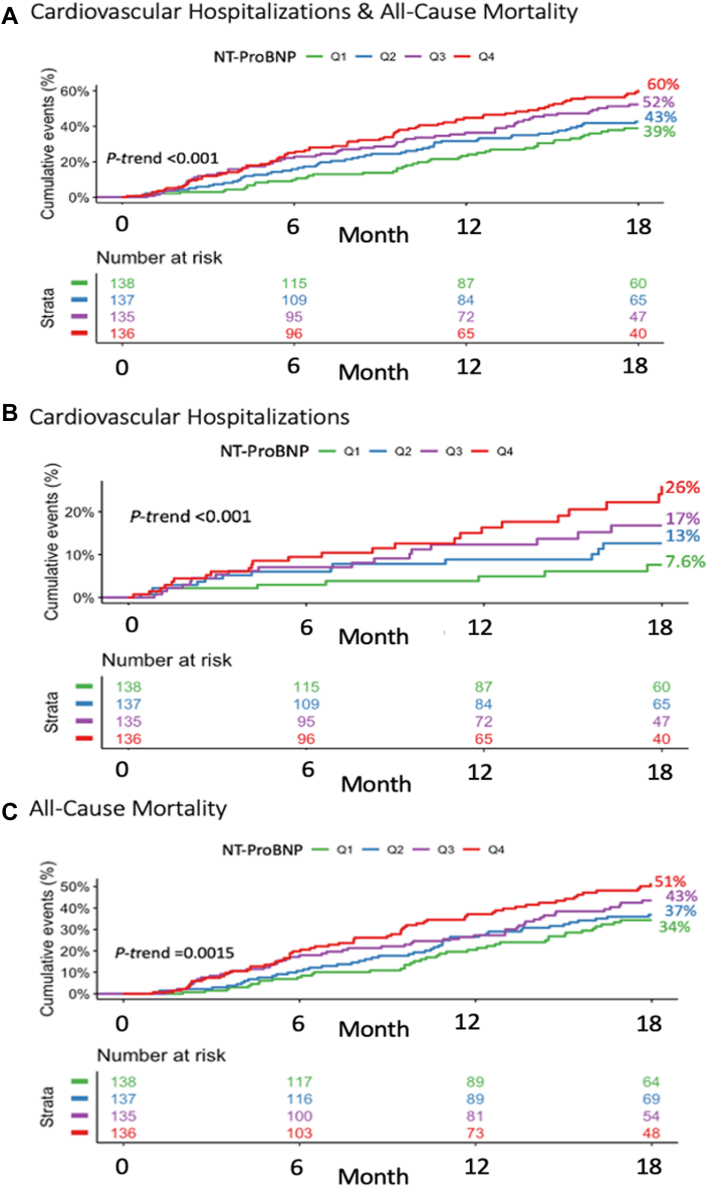
Kaplan-Meier Curves by N-Terminal Pro-B-Type Natriuretic Peptide Quartiles Kaplan-Meier curves for (A) the composite of all-cause death or cardiovascular hospitalization, (B) cardiovascular hospitalization, and (C) all-cause death stratified by quartiles of NT-proBNP. NT-proBNP = N-terminal pro-B-type natriuretic peptide.

**Figure 3:**
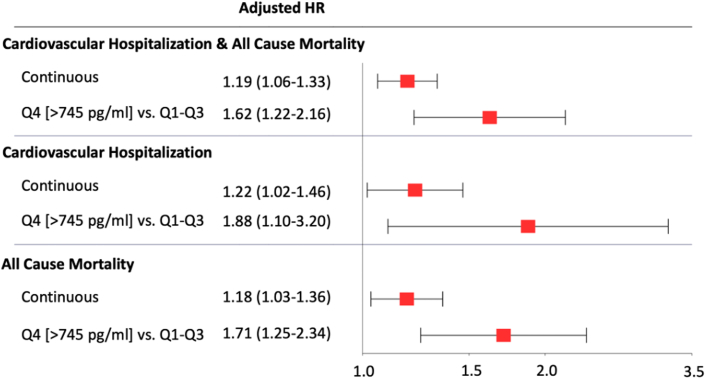
N-Terminal Pro-B-Type Natriuretic Peptide and Risk of Cardiovascular Events Association between NT-proBNP levels, analyzed as a continuous variable and as quartile (Q) 4 vs Q1–3, and the risk of cardiovascular hospitalization and death. Cox regression models were adjusted for age, sex, estimated glomerular filtration rate (eGFR), diabetes, coronary artery disease, heart failure, hypertension, atrial fibrillation, C-reactive protein, and low-density lipoprotein cholesterol. NT-proBNP = N-terminal pro-B-type natriuretic peptide.

As compared to patients with NT-proBNP concentrations <125 pg/mL, those with levels ≥125 pg/mL had significantly higher 12-month KM event rates of the composite endpoint of cardiovascular hospitalizations or death (39% vs 21%, *P* < 0.001) as well as for the individual components (both *P* < 0.01) (Central Illustration). There was a graded increase in risk of cardiovascular hospitalizations and death across quartiles of NT-proBNP (Q1: 22%, Q2: 28%, Q3: 29%, and Q4: 38%; *P*-trend <0.001) (Figure 2, Supplemental Figure 2). After multivariable adjustment, NT-proBNP, both modeled as a continuous variable (adjusted HR for 1-unit increase in standardized log-transformed biomarker: 1.19; 95% CI: 1.06-1.33) or as quartiles, remained independently associated with cardiovascular hospitalizations and death (Q4 vs Q1-Q3: adjusted HR: 1.62; 95% CI: 1.22-2.16) as well as for the individual components of the composite endpoint (Figure 3).

**Central Illustration:**
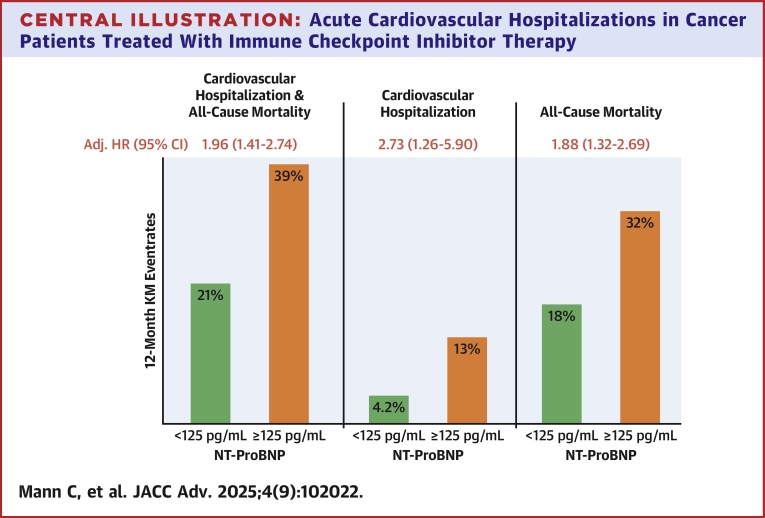
Acute Cardiovascular Hospitalizations in Cancer Patients Treated With Immune Checkpoint Inhibitor Therapy Kaplan-Meier event rates at 12 months and adjusted HRs for (A) the composite of all-cause death or cardiovascular hospitalization, (B) cardiovascular hospitalization, and (C) all-cause death stratified by an NT-proBNP threshold of 125 pg/mL. Cox regression models were adjusted for age, sex, estimated glomerular filtration rate (eGFR), diabetes, coronary artery disease, heart failure, hypertension, atrial fibrillation, C-reactive protein, and low-density lipoprotein cholesterol. KM = Kaplan-Meier; NT-proBNP = N-terminal pro-B-type natriuretic peptide.

**Figure 4:**
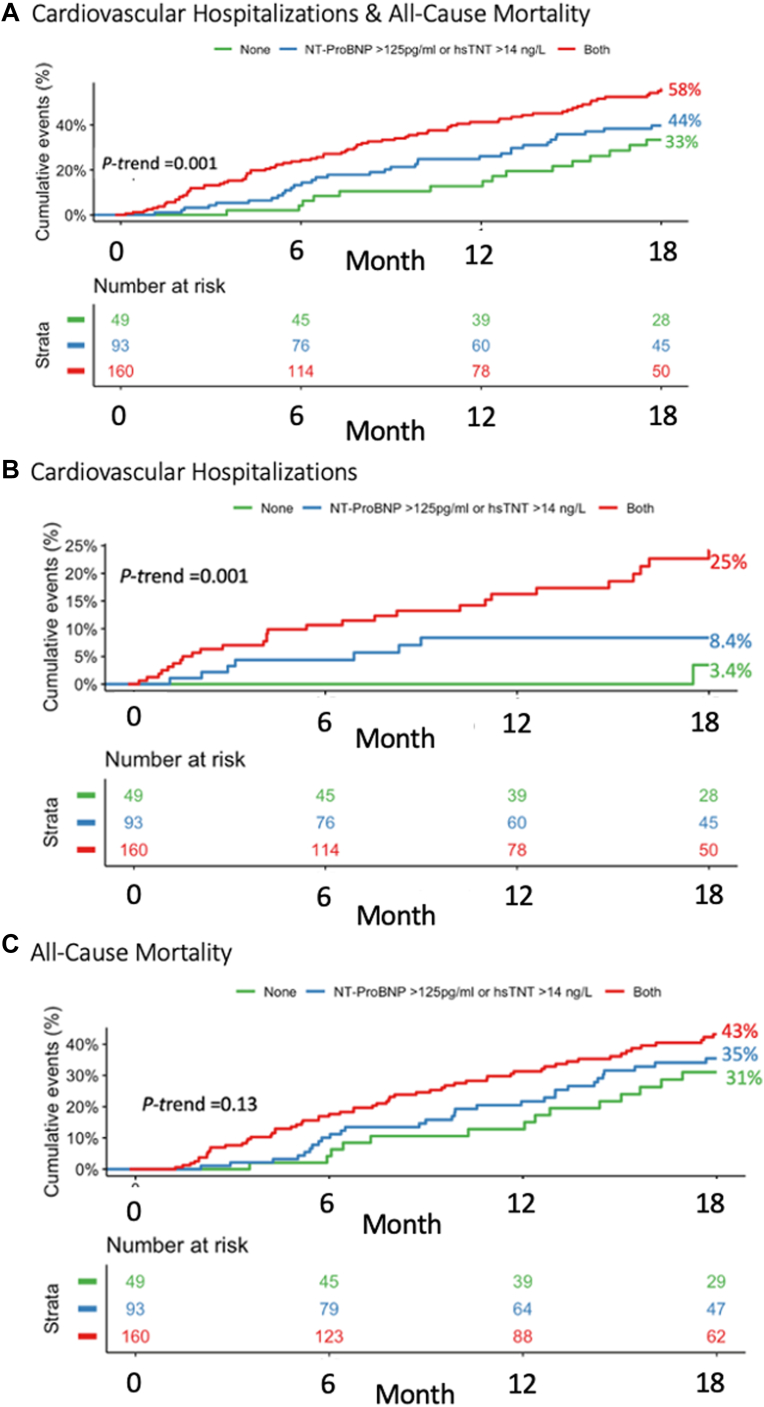
Kaplan-Meier Curves by N-Terminal Pro-B-Type Natriuretic Peptide and High-Sensitivity Troponin T Levels Kaplan-Meier curves for (A) the composite of all-cause death or cardiovascular hospitalization, (B) cardiovascular hospitalization, and (C) all-cause death stratified by levels of NT-proBNP and hsTnT (no increase in biomarkers, either NT-proBNP ≥125 pg/mL or hsTnT ≥14 ng/L, and both NT-proBNP ≥125 pg/mL and hsTnT ≥14 ng/L). hsTnT = high-sensitivity troponin T; NT-proBNP = N-terminal pro-B-type natriuretic peptide.

A similar relationship was observed for hsTnT concentrations. Patients with hsTnT levels <14 ng/L vs ≥14 ng/L had significantly higher rates of acute cardiovascular hospitalizations and death (12-month KM event rate: 37% vs 45%, *P*-log-rank = 0.015) (Supplemental Figure 3). Moreover, in a multimarker model (including both NT-proBNP and hsTnT), there was a stepwise increase in risk across the number of increased biomarkers (Figure 4 and Supplemental Figure 4). Specifically, those patients (16.2%) who had both biomarkers below the established threshold (hsTnT <14 ng/L and NT-proBNP <125 pg/mL) were at very low risk for acute cardiovascular events (<0.1% at 12 months).

## Discussion

In the present study, we found that cancer patients treated with ICI are at high risk for acute cardiovascular hospitalizations that were predominantly attributed to atherosclerosis and heart failure and that widely available cardiac biomarkers can be useful in detecting those at highest risk.

Our findings are consistent with numerous studies that have repeatedly shown elevated rates of cardiovascular events in cancer patients treated with ICIs.^3^^,^^9^^,^^27^^,^^28^ However, this increased risk is not solely attributed directly to the adverse effects of ICIs but reflects shared risk factors and overlapping biological mechanisms that contribute to both cancer progression and cardiovascular disease, thus thereby reflecting the underlying severity of cancer.^11^^,^^12^ We found that the predominant causes of hospitalization in our cohort were atherosclerosis and heart failure. Notably, no hospitalizations were explicitly attributed to myocarditis, likely reflecting the overall low incidence of severe immune-related myocarditis. However, cases of acute or subclinical myocarditis, as well as those managed in the outpatient setting, may have been underreported or not captured, which could have implications for patients' cardiovascular health and overall prognosis.^29^^,^^30^ Additionally, acute myocarditis could have been misclassified as acute heart failure, as differentiating between inflammatory and noninflammatory heart failure can be complex and diagnostically challenging, particularly due to the frequent absence of definitive findings on cardiac magnetic resonance imaging, even in cases of ongoing immune-related myocarditis.^31^ However, endomyocardial biopsy is not consistently part of standard practice across all hospitals.

Given the high incidence of cardiovascular events and the expanding use of these treatments, these data highlight the critical need for proactive cardiovascular risk stratification in cancer patients undergoing ICI therapy. We found that NT-proBNP concentrations were strongly associated with acute cardiovascular hospitalizations and mortality in cancer patients treated with ICIs, suggesting that NT-proBNP may serve as a helpful tool for identifying patients at high risk. The integration of NT-proBNP as a routine biomarker in the clinical management of cancer patients receiving ICIs could lead to earlier identification of those at highest cardiovascular risk, enabling early intervention strategies, including closer monitoring, lifestyle modification, and, in certain cases, the prophylactic use of cardioprotective medications. NT-proBNP, along with clinical characteristics, could also help tailor the intensity and frequency of cardiovascular monitoring. Furthermore, our study suggests that a multimarker approach combining NT-proBNP with hsTnT may be effective in identifying patients at an even greater risk for adverse cardiovascular outcomes, refining risk prediction, and thereby potentially guiding clinical decisions more effectively. Importantly, when both biomarkers are below their respective thresholds, the risk for cardiovascular hospitalizations is markedly reduced, possibly allowing more streamlined patient management by targeting preventive and monitoring efforts toward higher-risk patients, while low-risk individuals can avoid unnecessary interventions.

These findings are particularly relevant in the context of the 2022 ESC Guidelines on Cardio-Oncology, which recommend routine baseline biomarker assessment and serial follow-up screening in all cancer patients to identify patients at risk of cancer therapy-related cardiac dysfunction.^19^^,^^20^ However, the evidence supporting the use of cardiac biomarkers for risk stratification in ICI-treated cancer patients is limited and primarily based on a nested biomarker study of the JAVELIN-RENAL 101 trial^14^ that compared avelumab plus axitinib with sunitinib in patients with renal cell carcinoma. This study found that ICI-treated patients with high baseline troponin T (but not NT-proBNP) concentrations were at a higher risk of major adverse cardiovascular events. However, baseline troponin and NT-proBNP concentrations were only available for 209 and 131 patients, respectively. Additionally, patients with a reduced left ventricular ejection fraction were excluded, resulting in a low proportion of patients with increased NT-proBNP and hsTnT levels and a small number of events. The present study, thus, provides support for the 2022 ESC Guidelines on Cardio-Oncology, affirming NT-proBNP and hsTnT as valuable tools for risk stratification in a real-world cohort of ICI-treated cancer patients. However, to fully integrate these biomarkers into routine clinical practice, randomized controlled trials are urgently needed to evaluate targeted interventions in high-risk patients identified by these biomarkers. Moreover, their utility in on-treatment surveillance across different risk groups should be assessed, taking into account the economic impact of early and serial biomarker screening.

### Study limitations

First, this was a retrospective analysis, and despite adjusting for multiple variables, residual confounding is possible. Second, this study was limited by its single-center design at a tertiary academic center, and at that time, baseline and serial biomarkers were not measured in a standardized manner, leading to incomplete biomarker data and introducing potential selection bias. While we restricted our analysis to NT-proBNP measurements obtained within 3 months prior to ICI initiation, we cannot entirely exclude the possibility that some values were influenced by acute conditions. However, we sought to minimize this risk by excluding patients with documented acute cardiovascular events at the time of measurement. This study included only NT-proBNP measurements performed in-hospital, excluding values obtained in ambulatory or external laboratories. However, patient characteristics and event rates were similar between those with and without available NT-proBNP values, suggesting that missing data did not introduce systematic bias. As only a subset of patients received dual ICI therapy, our study was not powered to detect differences in cardiovascular outcomes between monotherapy and combination therapy. Additionally, the causes of hospitalizations were not adjudicated, and therefore, inaccurate diagnoses as well as underreporting are possible. We were also unable to adjudicate the causes of death, as we relied on documented death certificates. However, given the challenges in differentiating cardiovascular and non-cardiovascular death in this population and the competing risk of mortality, all-cause death was included as a clinically and methodologically relevant endpoint.

## Conclusions

Cancer patients treated with ICI are at high risk for acute cardiovascular hospitalizations that were predominantly attributed to atherosclerosis and heart failure. These findings affirm NT-proBNP and hsTnT as valuable tools for risk stratification in a vulnerable patient population facing the dual burden of malignancy and cardiovascular disease.

## Funding support and author disclosures

Dr Hülsmann has received research grants, honoraria for lectures or advisory boards, and educational grants from Roche Diagnostics, AstraZeneca, Biopeutics, CSL Vifor, Boehringer Ingelheim, Zoll, Bayer, Merck, Novartis, Astropharma, Pfizer, Bristol Myers Squibb, and Boston Scientific. Dr Zelniker has received research grants from the 10.13039/501100002428Austrian Science Funds, the German Research Foundation, and Boehringer Ingelheim; honoraria for serving on advisory boards from Boehringer Ingelheim and Bayer AG; personal fees from Alkem Lab. Ltd, AstraZeneca, Bayer AG, Boehringer Ingelheim, and Sun Pharmaceutical Industries; and educational grants from Eli Lilly and Company. All other authors have reported that they have no relationships relevant to the contents of this paper to disclose.

Perspectives **COMPETENCY IN MEDICAL KNOWLEDGE:** This study highlights the significant cardiovascular risks in ICI-treated cancer patients, particularly hospitalizations related to atherosclerosis and heart failure, and underscores the utility of cardiac biomarkers as key biomarkers for risk stratification.

**TRANSLATIONAL OUTLOOK:** Despite guideline recommendations, the evidence supporting the use of cardiac biomarkers for risk stratification in ICI-treated patients has been limited and inconclusive. This study provides critical evidence supporting the integration of NT-proBNP into clinical workflows, aligning with current cardio-oncology guidelines. Future randomized trials will be essential to validate biomarker-guided interventions.
