# Supplementary material for: Prognostic Value of Biomarkers in Cancer Patients Treated With Immune Checkpoint Inhibitor Therapy
Source: JACC Adv. 2025 Jul 31;4(9):102022. doi: 10.1016/j.jacadv.2025.102022 (PMC12335986; doi:10.1016/j.jacadv.2025.102022)
Supplement: Supplementary data [file mmc1.docx]

# Supplemental Appendix

## Supplemental methods

A systematic exploration of the centralized patient management system of the Vienna General Hospital (“*AKH Informationsmanagement*”) was done using the ICD codes stated below. Moreover, all outcomes were determined using the ICD codes reported on the death certificate or the admission diagnosis in the Vienna Healthcare Group hospitalizations database.

Cardiovascular Hospitalizations:

I05.0, I05.9, I08.0, I08.1, I08.2, I08.3, I10, I11.0, I11.9, I13.1, I13.2, I13.9, I21.0, I21.1, I21.3, I21.4, I21.9, I25.1, I25.5, I25.8, I25.9, I33.0, I34.0, I34.2, I34.8, I34.9, I35.0, I35.1, I35.2, I35.8, I35.9, I36.1, I36.9, I38, I42.0, I42.1, I42.2, I42.9, I45.1, I46.9, I47.2, I48.1, I48.9, I49.0, I49.9, I50.1, I50.9, I51.4, I51.5, I51.6, I51.7, I51.9, I63.9, I64, I67.2, I67.8, I67.9, I69.4, I70.2, I70.9, I71.0, I71.2, I71.3, I73.9

## Supplemental Tables

**Table 1: Baseline characteristics stratified by the availability of NT-proBNP**

Continuous data are reported as median (interquartile range).

Legend: CRP, C-reactive protein; eGFR, estimated glomerular filtration rate; hsTnT, high sensitivity Troponin T; LDL-C, low-density lipoprotein cholesterol

|  | **Overall**  **(n = 1142)** | **No NT-proBNP available**  **(n = 592)** | **NT-proBNP available**  **(n = 550)** | **P-value** |
| --- | --- | --- | --- | --- |
| **Age, years** | 64 (56, 72) | 63 (55, 71) | 65 (56, 73) | 0.085 |
| **Female sex, n (%)** | 449 (39%) | 256 (43%) | 193 (35%) | 0.005 |
| **Diabetes Mellitus, n (%)** | 113 (9.9%) | 42 (7.1%) | 71 (13%) | 0.001 |
| **Hypertension, n (%)** | 181 (16%) | 64 (11%) | 117 (21%) | <0.001 |
| **Atrial Fibrillation, n (%)** | 28 (2.5%) | 4 (0.7%) | 24 (4.4%) | <0.001 |
| **Heart Failure, n (%)** | 10 (0.9%) | 2 (0.3%) | 8 (1.5%) | 0.056 |
| **Coronary Artery Disease, n (%)** | 44 (3.9%) | 13 (2.2%) | 31 (5.6%) | 0.003 |
| **hsTnT, ng/L** | 16 (10, 30) | 14 (9, 29) | 16 (10, 31) | 0.15 |
| **eGFR, ml/min/1.73 m^2** | 77 (52, 108) | 73 (48, 103) | 81 (56, 112) | <0.001 |
| **CRP, mg/dL** | 1.0 (0.3, 3.5) | 0.9 (0.3, 3.5) | 1.1 (0.3, 3.3) | 0.7 |
| **HbA1c, %** | 5.70 (5.40, 6.20) | 5.90 (5.50, 6.65) | 5.70 (5.30, 6.10) | 0.005 |
| **LDL-C, mg/dL** | 96 (72, 123) | 101 (77, 125) | 95 (66, 122) | 0.11 |

**Table 2: Underlying Malignant Disease**

|  | **NT-proBNP Population**   (n = 550) |  | **hsTnT Population**   (n = 305) |
| --- | --- | --- | --- |
| **Melanoma and other malignant neoplasms of skin, n(%)** | 74 (13%) |  | 65 (21%) |
| **Malignant neoplasms of respiratory and intrathoracic organs, n(%)** | 130 (24%) |  | 4 (1.3%) |
| **Malignant neoplasms of eye, brain and other parts of central nervous system, n(%)** | 12 (2.2%) |  | 8 (2.6%) |
| **Malignant neoplasm of breast, n(%)** | 16 (2.9%) |  | 34 (11%) |
| **Malignant neoplasms of digestive organs, n(%)** | 57 (10%) |  | 20 (6.6%) |
| **Malignant neoplasms of lip, oral cavity and pharynx, n(%)** | 36 (6.5%) |  | 5 (1.6%) |
| **Malignant neoplasms of bone and articular cartilage, n(%)** | 8 (1.5%) |  | 3 (1.0%) |
| **Malignant neoplasms of mesothelial and soft tissue, n(%)** | 6 (1.1%) |  | 6 (2.0%) |
| **Malignant neoplasms of female genital organs, n(%)** | 8 (1.5%) |  | 8 (2.6%) |
| **Malignant neoplasms of male genital organs, n(%)** | 11 (2.0%) |  | 49 (16%) |
| **Malignant neoplasms of urinary tract, n(%)** | 79 (14%) |  | 2 (0.7%) |
| **Malignant neoplasms of thyroid and other endocrine glands, n(%)** | 2 (0.4%) |  | 88 (29%) |
| **Benign neoplasms, n(%)** | 169 (31%) |  | 88 (34% |
| **Neoplasms of uncertain or unknown behaviour, n(%)** | 169 (35%) |  | 55 (18%) |

**Table 3: Prescribed Immune Checkpoint Inhibitor**

|  | **NT-proBNP Population**  (n = 550) | **hsTnT Population**  (n = 305) |
| --- | --- | --- |
| **Monotherapy, n(%)** | 487 (88.5%) | 280 (91.8%) |
| **Pembrolizumab, n(%)** | 303 (55%) | 152 (50%) |
| **Nivolumab, n(%)** | 184 (33%) | 127 (42%) |
| **Atezolizumab, n(%)** | 79 (14%) | 37 (12%) |
| **Avelumab, n(%)** | 15 (2.7%) | 5 (1.6%) |
| **Cemiplimab, n(%)** | 8 (1.5%) | 5 (1.6%) |
| **Ipilimumab, n(%)** | 71 (13%) | 52 (17%) |
| **Durvalumab, n(%)** | 16 (2.9%) | 4 (1.3%) |

**Table 4: Baseline characteristics stratified by NT-proBNP quartiles**

Continuous data are reported as median (interquartile range). CRP, C-reactive protein; eGFR, estimated glomerular filtration rate; hsTnT, high sensitive Troponin T; LDL-C, low-density lipoprotein cholesterol; Q, quartile

|  | **Overall**  (n = 550) | **NT-proBNP Quartiles** | | | |
| --- | --- | --- | --- | --- | --- |
|  |  | **Q1**  **(<102 pg/mL)**  (n = 138) | **Q2**  **(102-271 pg/mL)**  (n = 138) | **Q3**  **(271-742 pg/mL)**  (n = 137) | **Q4**  **(>742 pg/mL)**  (n = 137) |
| **NT-proBNP, pg/mL** | 271 (102, 742) | 51 (33, 73) | 175 (136, 226) | 446 (360, 577) | 1,668 (1,045, 4,373) |
| **Age, years** | 65 (56, 73) | 58 (50, 63) | 63 (56, 72) | 69 (60, 75) | 70 (63, 76) |
| **Female sex, n (%)** | 193 (35%) | 47 (34%) | 53 (38%) | 42 (31%) | 51 (37%) |
| **Diabetes Mellitus, n (%)** | 71 (13%) | 17 (12%) | 15 (11%) | 14 (10%) | 25 (18%) |
| **Hypertension, n (%)** | 117 (21%) | 22 (16%) | 29 (21%) | 37 (27%) | 29 (21%) |
| **Atrial Fibrillation, n (%)** | 24 (4.4%) | 0 (0%) | 2 (1.4%) | 5 (3.6%) | 17 (12%) |
| **Heart Failure, n (%)** | 8 (1.5%) | 0 (0%) | 1 (0.7%) | 2 (1.5%) | 5 (3.6%) |
| **Coronary Artery Disease, n (%)** | 31 (5.6%) | 1 (0.7%) | 4 (2.9%) | 11 (8.0%) | 15 (11%) |
| **hsTnT available, n (%)** | 305 (55%) | 59 (43%) | 65 (47%) | 77 (56%) | 104 (76%) |
| **hsTnT, ng/L** | 16 (10, 31) | 9 (6, 15) | 15 (10, 23) | 17 (11, 27) | 26 (16, 44) |
| **hsTnT ≥14 ng/L, n(%)** | 185 (61%) | 18 (31%) | 34 (52%) | 47 (61%) | 86 (83%) |
| **eGFR, ml/min/1.73 m^2** | 81 (56, 112) | 83 (63, 104) | 75 (50, 101) | 80 (56, 109) | 91 (68, 133) |
| **CRP, mg/dL** | 1.1 (0.3, 3.3) | 0.5 (0.2, 1.3) | 1.2 (0.4, 3.5) | 1.4 (0.5, 4.3) | 1.4 (0.4, 4.1) |
| **HbA1c, %** | 5.70 (5.30, 6.10) | 5.70 (5.20, 6.03) | 5.70 (5.40, 6.10) | 5.60 (5.30, 6.00) | 5.60 (5.40, 6.40) |
| **LDL-C, mg/dL** | 95 (66, 122) | 116 (87, 138) | 96 (70, 111) | 90 (66, 116) | 80 (54, 104) |

**Table 5: Baseline characteristics stratified by the increase of NT-proBNP** **≥125 pg/ml and/or hsTnT ≥14ng/L**.

Continuous data are reported as median (interquartile range). CRP, C-reactive protein; eGFR, estimated glomerular filtration rate; hsTnT, high sensitive Troponin T; LDL-C, low-density lipoprotein cholesterol; Q, quartile

|  | **Total Cohort**  (n = 305) | **Increased Biomarkers** | | |
| --- | --- | --- | --- | --- |
|  |  | **None**  (n = 49) | **One**  (n = 94) | **Two**  (n = 162) |
| **Age, years** | 67 (58, 74) | 58 (52, 65) | 64 (55, 70) | 71 (63, 76) |
| **Female sex, n (%)** | 98 (32%) | 15 (31%) | 37 (39%) | 46 (28%) |
| **Diabetes Mellitus, n (%)** | 47 (15%) | 6 (12%) | 11 (12%) | 30 (19%) |
| **Hypertension, n (%)** | 81 (27%) | 11 (22%) | 19 (20%) | 51 (31%) |
| **Atrial Fibrillation, n (%)** | 22 (7.2%) | 0 (0%) | 3 (3.2%) | 19 (12%) |
| **Heart Failure, n (%)** | 6 (2.0%) | 0 (0%) | 1 (1.1%) | 5 (3.1%) |
| **Coronary Artery Disease, n (%)** | 27 (8.9%) | 1 (2.0%) | 4 (4.3%) | 22 (14%) |
| **NT-proBNP, pg/mL** | 393 (136, 1,071) | 51 (34, 76) | 277 (128, 583) | 799 (359, 1,964) |
| **NT-proBNP ≥125pg/mL, n (%)** | 233 (76%) | 0 (0%) | 71 (76%) | 162 (100%) |
| **hsTnT, ng/L** | 17 (10, 31) | 8 (6, 10) | 10 (8, 13) | 28 (20, 41) |
| **hsTnT ≥14ng/L, n (%)** | 185 (61%) | 0 (0%) | 23 (24%) | 162 (100%) |
| **eGFR, ml/min/1.73 m^2** | 85 (62, 123) | 82 (60, 136) | 76 (50, 93) | 100 (72, 156) |
| **CRP, mg/dL** | 1.1 (0.3, 3.5) | 0.6 (0.2, 1.9) | 0.8 (0.2, 3.1) | 1.6 (0.5, 3.7) |
| **HbA1c, %** | 5.60 (5.30, 6.00) | 5.70 (5.40, 5.80) | 5.60 (5.20, 6.00) | 5.60 (5.30, 6.10) |
| **LDL-C, mg/dL** | 96 (69, 121) | 119 (91, 143) | 97 (71, 120) | 87 (61, 109) |

## Supplemental Figures

**Figure 1:** Number of cardiovascular hospitalizations per patient


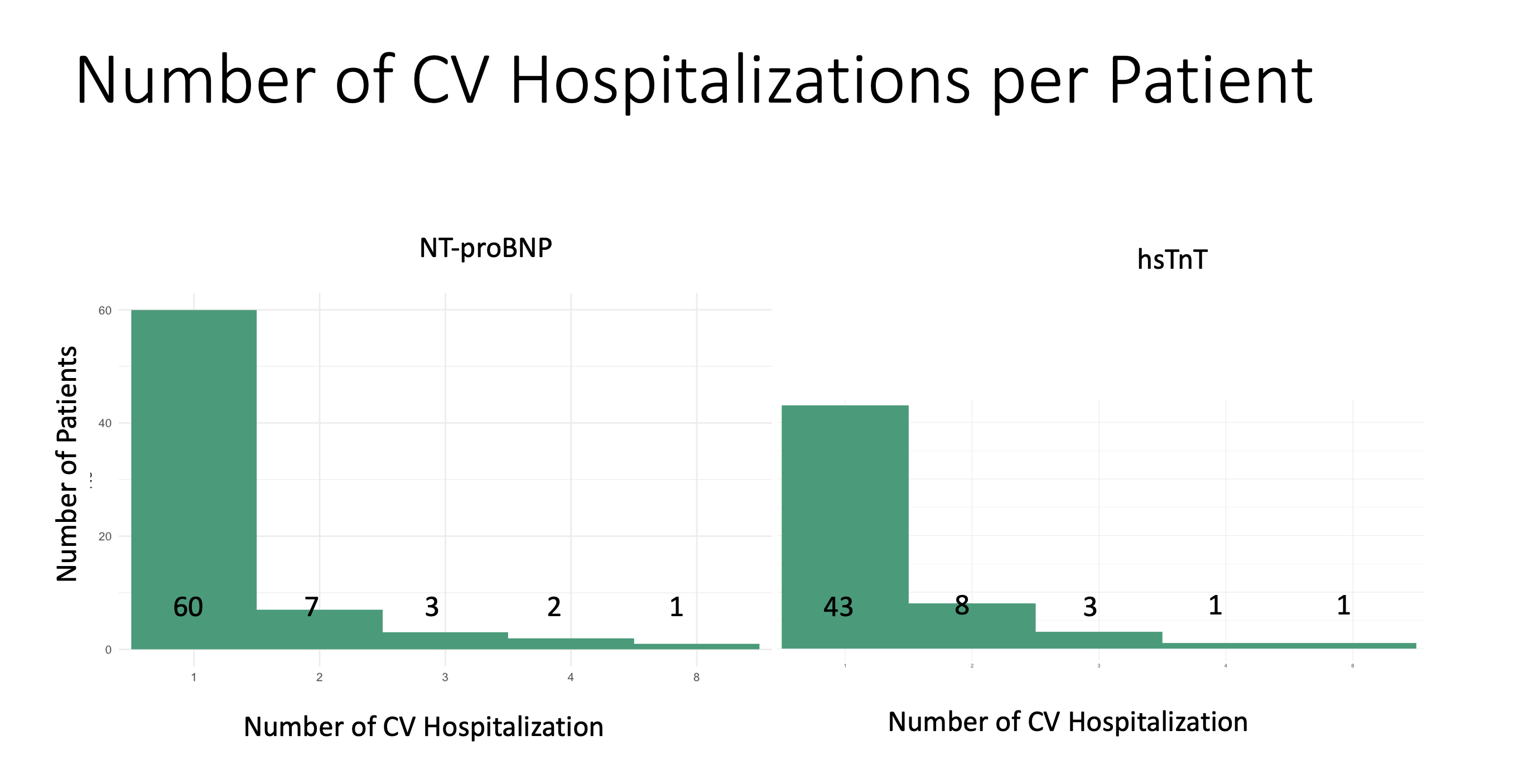


**Figure 2: Kaplan–Meier curves for (A) the composite of all-cause death or cardiovascular hospitalization, (B) cardiovascular hospitalization, and (C) all-cause death stratified by NT-proBNP Q4 vs. Q1-3**

**
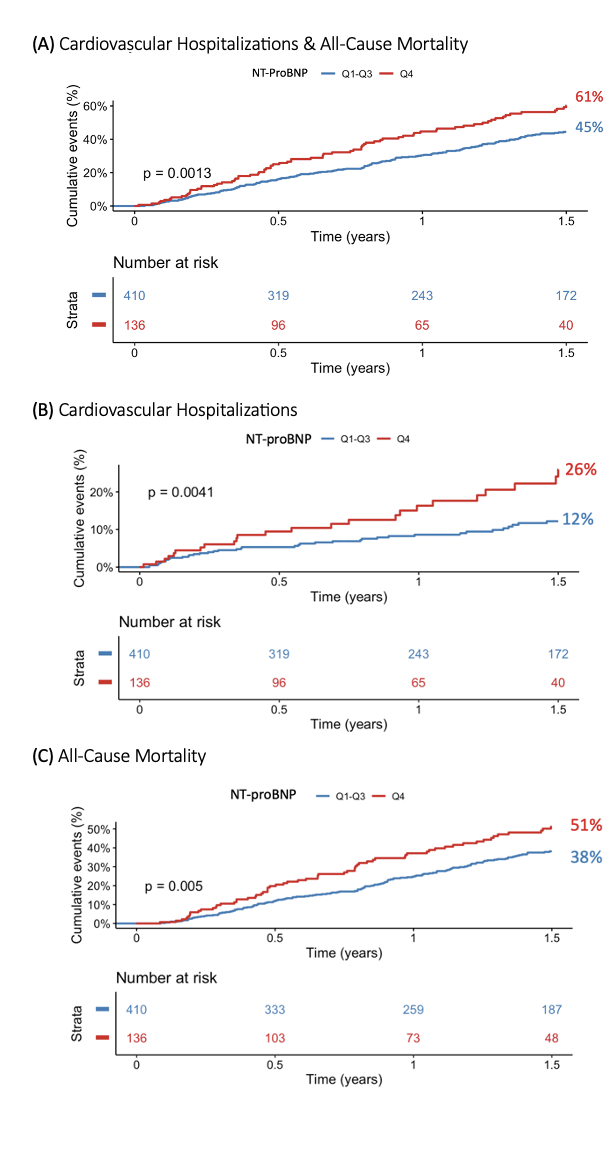
**

**Figure 3: Kaplan–Meier curves for (A) the composite of all-cause death or cardiovascular hospitalization, (B) cardiovascular hospitalization, and (C) all-cause death stratified by hsTnT <14 versus ≥14 ng/L.**

**
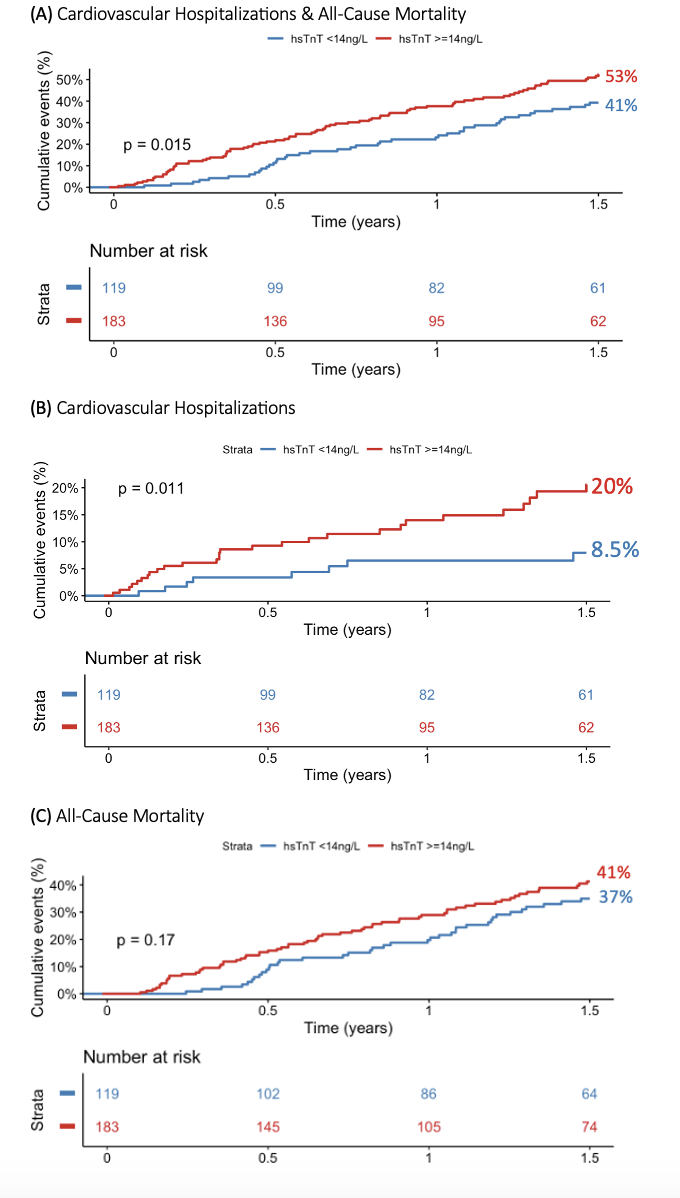
**

**Figure 4: Adjusted hazard ratios for the composite of all-cause death or cardiovascular hospitalization stratified by levels of NT-proBNP and hsTnT (no increase in biomarkers, either NT-proBNP ≥125 pg/ml or hsTnT ≥14 ng/L, and both NT-proBNP ≥125 pg/ml and hsTnT ≥14 ng/L).**

Cox regression models were adjusted for age, sex, creatinine, diabetes, heart failure, coronary artery disease, hypertension, atrial fibrillation, and C-reactive protein.

**
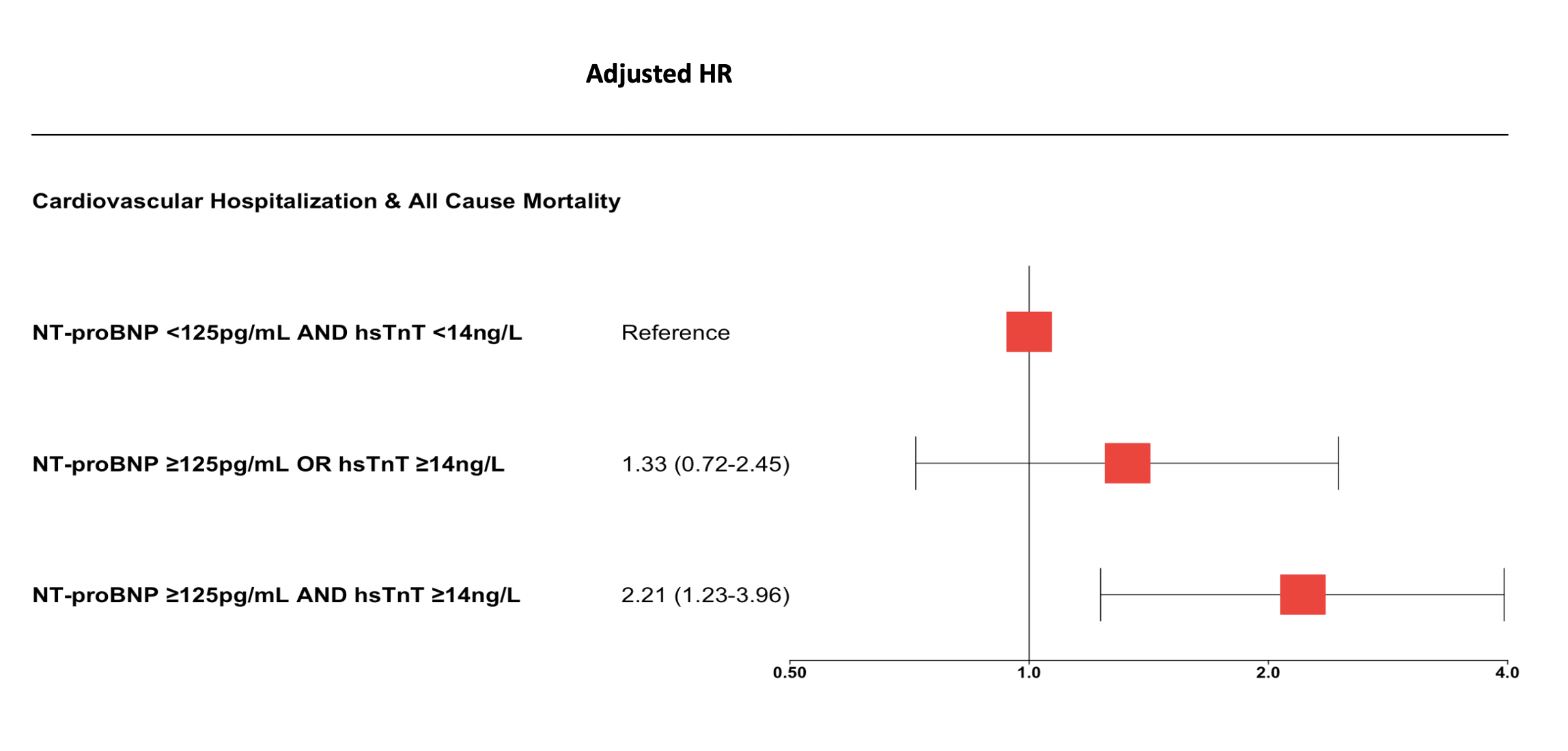
**
